# Aggressive behaviours associated with MDMA and psychedelics: a narrative review

**DOI:** 10.1017/neu.2024.3

**Published:** 2024-02-08

**Authors:** Negar Sayrafizadeh, Nicole Ledwos, M. Ishrat Husain, David J. Castle

**Affiliations:** 1 Centre for Complex Interventions, Centre for Addiction and Mental Health (CAMH), Toronto, ON, Canada; 2 Campbell Family Mental Health Research Institute, Centre for Addiction and Mental Health, Toronto, ON, Canada; 3 Department of Psychiatry, University of Toronto, Toronto, ON, Canada

**Keywords:** Psychedelics, aggression, violence, MDMA, hallucinogens

## Abstract

**Objective:**

Monoamine neurotransmitters play a role in aggression, especially when altered by illicit substances. However, some literature suggests that not all illicit substances may lead to aggression, notably psychedelics. This narrative review investigates the associations between serotonergic psychedelics and MDMA on aggressive behaviour.

**Methods:**

PubMed and PsycINFO were searched for original, peer-reviewed articles evaluating the effects of serotonergic psychedelics and 3,4-methyl enedioxy methamphetamine (MDMA) on violent and aggressive behaviour using Preferred Reporting Items for Systematic Reviews and Meta-Analyses (PRISMA) guidelines.

**Results:**

After removing duplicates, a total of 555 articles were screened, with 16 meeting the inclusion criteria. One additional article was obtained through reference screening bringing the total to 17 articles. Of these 17 articles, 14 studies focused on MDMA and three on serotonergic psychedelics. Findings were mixed, with some results demonstrating increased aggression following psychedelics and others suggesting protective effects. Limitations in the current literature include varied definitions of psychedelics, lack of standardised objective outcome measures and failure to control for confounding.

**Conclusion:**

As psychedelic research continues to expand, further assessment on the effects of serotonergic psychedelics and MDMA on aggressive behaviour is required.

Significant outcomes

- A brief history of psychedelic drugs, their different classes and their mechanism of action on aggressive behaviour are presented.
- There is a limited number of studies that investigate associations between psychedelic use and aggressive behaviour.
- The included studies share similar shortcomings that must be addressed in future research to improve understanding on associations between psychedelic use and aggressive behaviour.

Limitations

- This review included contemporary studies on associations between psychedelic use and aggression and does not include all available literature.
- This review excludes literature with indirect outcome variables related to violence or aggression.

## Introduction

Psychedelic drugs are most notoriously associated with the counterculture movement of the mid-twentieth century when reports of lysergic acid diethylamide (LSD) and ‘magic mushrooms’ (psilocybin) made their way into mainstream media. Plant-based psychedelics have been used for hundreds of years in indigenous communities for religious and ceremonial purposes (Garcia-Romeu *et al*., 2016). From the 1950s to the 1970s, some patients were treated using psychedelic drugs through clinical research trials (Garcia-Romeu *et al*., 2016; Rucke *et al*., 2018). Results from these early studies indicated that psychedelics might enhance traditional psychotherapy, with some participants demonstrating clinical improvement (Rucker *et al*., 2018). However, in the United States (US) in the 1970s, the Controlled Substances Act signed by the Nixon administration categorised psychedelics as Schedule 1 substances, halting most research (Carhart-Harris and Goodwin, 2017). In the late 1970s, 3,4-methylenedioxymethamphetamine (MDMA) started to gain popularity among psychiatrists due to the belief that patient insight and communication improved as a result of combining MDMA with therapy (Grinspoon and Bakalar, 1986); MDMA also started to become a popular recreational drug (Parrott, 2001; Passie, 2018). In 1985, the US Drug Enforcement Administration banned MDMA and placed it among Schedule 1 drugs (DEA 2018).

‘Psychedelic’ is an umbrella term for several drugs traditionally categorised by their neurobiological mechanism of action and chemical structure. So-called ‘classic’ psychedelics include serotonin receptor agonists such as LSD, psilocybin, peyote, ayahuasca and N,N-dimethyltryptamine (DMT) (Nichols, 1986; Garcia-Romeu *et al*., 2016) However, other pathways and receptors have been implicated in the mechanism of action for some of the drugs including mescaline and DMT. Evidence suggests that mescaline influences dopaminergic activity and 5-hydroxyindoleacetic acid (5-HIAA) levels in the brain (Dinis-Oliveira *et al*., 2019). DMT has been found to have an affinity for mGluR2 glutamate, sigma-1, the α1- and α2-adrenergic receptors, as well as the dopamine D1 receptor (Hamill *et al*., 2019). Substances such as 3,4-methylenedioxymethamphetamine (MDMA) that combine the catecholaminergic effects of methamphetamine with the serotonergic effects of psychedelics have been categorised as empathogens. Empathogens typically produce psychedelic-like effects without hallucinations (Nichols, 1986).

Classic psychedelics, by definition, are 5-HT_2A_ receptor agonists that act primarily as full or partial agonists of the serotonin (5-HT) 2A receptors (Nichols, 2016). Several studies suggest that classic psychedelics increase communication between multiple disparate brain regions while decreasing activity in the default mode network (DMN) (Nichols, 2016). This pattern may explain the sense of unity and ego dissolution often associated with the psychedelic experience (Nichols, 2016). MDMA, on the other hand, acts through increasing release of serotonin, noradrenaline, and dopamine and is also associated with release of oxytocin, which might explain some of its empathogenic effect (Liechti *et al*., 2000; Dunlap *et al*., 2018). MDMA alters blood flow to brain regions involved in emotion formation, processing and behavioural learning and may be particularly important in regulating fear-based behaviours (Vollenweider, 2022). Taken together, these drugs impact several areas of the brain that play central roles in human behaviour and emotional processing. However, there are mixed findings regarding the association between psychedelic use and aggression/violence. The most frequent explanation for violence and aggression is related to low serotonin levels and, as mentioned earlier, this is not found to be a neurobiological effect of classical psychedelics (Boles and Miotto, 2003). Instead, feelings of invincibility and ego dissolution can be part of the psychedelic experience (Nichols, 2016). These feelings of invincibility may explain some users’ aggressive or violent outbursts following use. On the other hand, there is some evidence to suggest that, although hallucinogenic substances do not usually trigger violent behaviours per se, they may exacerbate the effects of underlying psychopathologies, which can lead to aggression; this has been described in studies of LSD, for example (Reiss and Roth, 1993). On a similar note, there is evidence to suggest that paranoid ideation can increase violent behaviour (Mojtabai, 2006; Coid *et al*., 2016). Given that paranoia is found to be one of the side effects of psychedelic use (Schlag *et al*., 2022), it can be inferred that this side effect could possibly result in violent behaviour in users.

As research into the therapeutic use of psychedelics expands, the association between these compounds and violent or aggressive behaviour is important to consider especially given the mixed findings mentioned above. Substance use is frequently involved in violent behaviour, with a substantial proportion of incarcerated violent offenders being substance-involved (Boles and Miotto, 2003). Alcohol, cocaine and phencyclidine (PCP) may trigger violent behaviour (Boles and Miotto, 2003). Alcohol, cocaine and psychedelics, among others, impact the monoamine neurotransmitters (serotonin, dopamine, norepinephrine) involved in behaviour regulation. However, the link between psychoactive substances and behaviour differ by drug type, the amount and pattern of use, as well as individual characteristics such as personality. For instance, chronic use of illicit substances has been found to alter the normal functioning of the nervous system, effects that can impact social communication and emotional processing; such factors could underpin aggressive behaviours (Boles and Miotto, 2003).

Furthermore, pre-existing aggressive conditions in childhood, prior to the onset of substance use, may also explain why some individuals’ aggressive and violent tendencies are exacerbated following substance use (Boles and Miotto, 2003). The prediction of violent behaviour in the context of substance use is highly complex, given the multiple contexts (e.g. environmental, social, situational and cultural) that impact violent outcomes as well as inter-individual differences in physiology, psychology, personality, personal history, sex and gender (Boles and Miotto, 2003).

There are also many methodological constraints in studies of substance use and aggression/violence. For one, illicit substances are often grouped together and/or used together, making it difficult to draw conclusions about the effects specific substances (Boles and Miotto, 2003). Furthermore, various stages of substance use, such as acute intoxication, withdrawal and drug-seeking behaviour, may lead to different types of violence and aggression (Boles and Miotto, 2003).

The primary aim of this narrative review is to investigate the current literature on the associations between psychedelics and violent and aggressive behaviours and to explore potential mechanisms for any associations. We included all classic psychedelics and MDMA, as these are drugs of current interest in psychiatry (Nutt and Castle 2022).

## Methods

### Search strategy

As this is a narrative review, only PubMed and PsycINFO databases were searched. We included peer-reviewed, original studies written in English published between the years 2000 through to the end of July 2022. This period was chosen because it covers the ‘modern’ era of research involving psychedelic agents. The following search terms were employed: ‘psychedelic*’ OR ‘MDMA’ OR ‘3,4-Methylenedioxymethamphetamine’ OR ‘Psilocybin’ OR ‘Psiloci’ OR ‘LSD’ OR ‘Lysergic acid diethylamide’ OR ‘Mescaline’ OR ‘DMT’ OR ‘N,N-Dimethyltryptamine’ OR ‘5-MeO-DMT’ OR ‘Ayahuasca’ AND ‘Aggressive behavior?r’ OR ‘Violent behavior?r’ OR ‘Criminal behavior?r’ OR ‘Aggress*’ OR ‘Violen*’. These keywords were searched along with relevant search terms adapted to each database. Reference lists of included articles were checked for any publications that might have been missed by our search strategy.

### Inclusion and exclusion criteria

Following the PICO framework, the criteria for selecting the studies were as follows: population (P): Participants 16 years of age or older with a history of lifetime psychedelic use; Intervention (I): Psychedelic use; Comparison (C): No comparison or studies including groups of participants with other substance use disorders or a control group which includes participants with no substance use disorder; Outcomes (O): Any form of aggressive or violent behaviour (e,g., physical, verbal, sexual) as either the primary or secondary outcome, established using validated measures (e.g. Conflicts Tactics Scale) or incarceration records.

The exclusion criteria were: 1) studies that did not include a group of participants with lifetime psychedelic use or were individuals<16 years of age; 2) abstracts for ongoing trials, conference abstracts, dissertations, opinions and commentaries. There were no restrictions on setting or language, and efforts were made to locate an English version of articles published in a different language.

### Data extraction

All studies generated from the search were uploaded into the Covidence software (Veritas Health Innovation, 2022), where duplicate studies were automatically removed. Two independent reviewers (N.S. & N.L.) screened the titles and abstracts for all studies against the pre-defined inclusion and exclusion criteria. Both reviewers obtained and reviewed full texts for all selected studies (N.S. & N.L.). Any conflicts at the title/abstract screening stage and the full-text review stage were resolved by a third independent reviewer (DC). Data extraction was conducted by one reviewer (NS) and included the following information: 1) author and publication year; 2) study design; 3) sample size and demographic information; 4) type of psychedelic; 5) outcome measures; 6) study results. This review was registered on the Open Science Framework (DOI 10.17605/OSF.IO/HRNG7) database.

## Results

**Figure 1.**
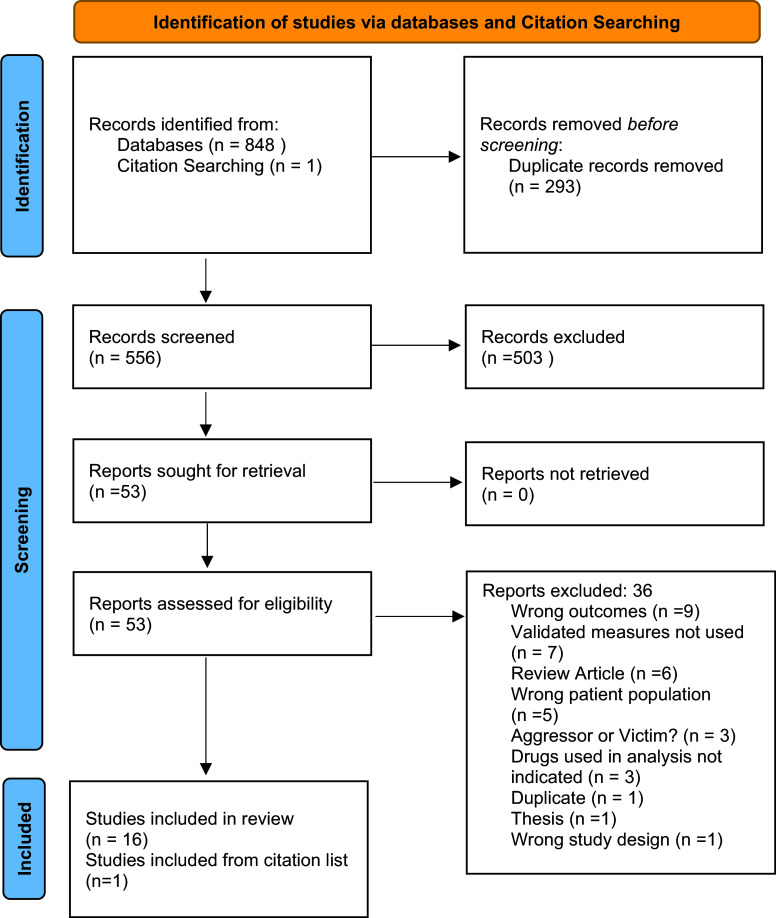
PRISMA 2020 flow diagram for new systematic reviews which included searches of databases and registers only. *From:* Page MJ, McKenzie JE, Bossuyt PM, Boutron I, Hoffmann TC, Mulrow CD, et al., The PRISMA 2020 statement: an updated guideline for reporting systematic reviews. BMJ 2021;372:n71. doi: 10.1136/bmj.n71. For more information, visit: http://www.prisma-statement.org/.

The search identified a total of 848 studies. Following the removal of 293 duplicates, 555 studies progressed to the title and abstract review phase (See Fig. 1). Of the 848 articles, 53 were selected for full-text screening and 16 met our eligibility criteria. By reviewing the citation list of one relevant study (Gerra *et al*., 2001), one additional study was found to be eligible, bringing the total number of included studies to 17. A complete summary of each trial is outlined in Table 1.

**Table 1.** Summary of trials

First Author, Year	Study Design	Sample Size and Demographic	Substance	Outcome Measure (s), Instruments of Measurement	Results
	**Hallucinogens/ classic psychedelics** (3 studies, *N* = 1718)				
Feingold *et al*., 2008	Longitudinal - focus on between participant associations	150 at-risk community sample of men in long-term relationships from late adolescence to the late 20s	Hallucinogens	Intimate partner violence, Adjustment with Partner Questionnaire (physical and psychological aggression), injuries through self-reports, partner reports, interviewer ratings and behavioural assessments.	The strongest predictors of IPV were marijuana and hallucinogen dependencies (even after controlling for antisocial behaviour)
Thiessen *et al*., 2018	Cross-Sectional	1266 community members	Classic Psychedelics	Intimate partner violence, revised short-form Conflicts Tactics Scale	Males only: lifetime history of psychedelic use inversely related to perpetration of IPV. Better emotion regulation observed among Psychedelic users
Walsh *et al*., 2016	Prospective	302 incarcerated men	Hallucinogens	Intimate partner violence, Arrest for an offence related to IPV. Coded based on the Law Enforcement Agencies Data System database.	Men who reported a lifetime history of hallucinogen use and lifetime presence of a hallucinogen use disorder were less likely to be arrested for perpetrating violence against an intimate partner than those with no history of hallucinogen use or disorder. Those who met criteria for hallucinogen use disorder, 90.90% (n = 20) reported lifetime use of classic psychedelics.
	**MDMA** (Studies: 14, *N* = 27,837 )				
Bond *et al*., 2004	A double-blind independent groups	96: 32 current MDMA users abstinent for 3 weeks, 32 ex-users abstinent for longer than 1 year and 32 non-MDMA substance users	MDMA	Trait anger, aggression, angry cognitive bias Multidimensional Anger Inventory, the Aggression Questionnaire Stories task	No group differences: All participants were faster to process the angry rather than the nonangry sentences. This relationship was strongest among current MDMA users Compared to controls, ex-users scored higher on trait aggression.
Curran *et al*., 2004	Independent group, repeated measures	61: 29 MDMA users and 32 controls	MDMA	Trait aggression, Aggressive Cognitive Bias The Aggression Rating Scale. The Aggression Questionnaire, Interpretative bias task	Compared to controls, on Day 0, MDMA users’ ratings were lower, relatively higher on day 4, and similar on day 7. Few days after ecstasy use: MDMA users responded faster to ambiguous aggressive than neutral sentences. The opposite was seen in the control group. No group differences in trait aggression (AQ) Controls took longer to recognise aggressive compared to neutral sentences; MDMA users showed the opposite pattern with faster reaction times to aggressive sentences
Gerra *et al*., 2000	Cross-Sectional	30: 15 MDMA users and 15 controls	MDMA	Aggression Buss-Durkee Hostility Inventory	At 3 weeks, compared to controls, ecstasy users had higher BDHI scores. At 12 months, MDMA users’ BDHI score reduced, and there were no significant group differences.
Gerra *et al*., 2001	Cross-Sectional	32: 12 MDMA users and 20 controls	MDMA	Aggression, Buss-Durkee Hostility Inventory, Point Subtraction Aggression Paradigm (behavioural aggression task)	Ecstasy users scored significantly higher on BDHI Direct Aggression and Irritability scales. They also showed significantly more aggressive responses across all three PSAP sessions than control subjects.
Hendrickson and Gerstein, 2005	Case-control	7794 arrested men in the 2001 Arrestee Drug Abuse Monitoring (ADAM) sample and 9764 male respondents of similar age in the 2001 National Household Survey on Drug Abuse (NHSDA).	Ecstasy	Criminal behaviour, FBI index offences and arrest history data in the Arrestee Drug Abuse Monitoring sample.	Ecstasy use negatively related to violent and property offences among arrestees. Compared to non-ecstasy users, ecstasy users were less likely to be charged with assault or burglary but were more likely to be charged with a drug-related offence.
Hoshi *et al*., 2004	Independent group, repeated measures	37: 16 ecstasy users and 21 controls	Ecstasy	Trait aggression Aggression Questionnaire, Aggression Rating Scale	Ecstasy users’ ARS ratings were lower than controls on day 0 but higher than controls on day 4. No significant difference was found between the groups on state measure of aggression Ecstasy users were more accurate than controls at recognising fear on day 0 and less accurate than controls on day 4. A greater amount of use and longer duration of use were associated with lower fear recognition accuracy scores on day 4.
Hoshi *et al*., 2006	Independent group, repeated measure	46: 19 ecstasy users and 27 controls	Ecstasy	Trait aggression, Aggressive Interpretative Bias Aggression Rating Scale, Aggression Questionnaire	Ecstasy users reacted slower to neutral sentences and recognised more disambiguated aggressive sentences than controls. Both groups showed similar reaction times to aggressive sentences and recognised similar amounts of neutral sentences. Ecstasy users rated themselves less aggressive and depressed than controls on day 0. But more depressed and aggressive on day 4. Among ecstasy users, no difference in aggression score between the two genders. Both genders showed aggressive bias towards interpreting ambiguous material.
Hoshi *et al*., 2007	Independent group	105: 26 ex-ecstasy users, 25 current ecstasy users, 29 polydrug-using controls and 25 drug-naive controls.	Ecstasy	Trait aggression and aggressive cognitive bias Aggression Questionnaire	No group differences in self-rated aggression. No evidence suggests that current or ex-ecstasy users have an aggressive interpretative bias.
Reid *et al*., 2007	Cross-sectional	260 ecstasy users	Ecstasy	Aggressive and violent behaviour Constructed an additive scale of four aggressive behaviours	A higher prevalence of lifetime ecstasy use is associated with higher levels of aggressive and violent behaviour. The odds of committing more violent acts in the past year have increased linearly with the number of ecstasy pills used. Greater lifetime ecstasy use has more of an impact on the aggressive behaviour of those with high self-control than those with low self-control. Those with high self-control exhibit much less aggression than those with low self-control at low levels of ecstasy use. But at high levels of lifetime ecstasy use, those with high self-control exhibit more aggression than those with low self-control. Males showed less aggressive behaviour compared to females when looking at the impact of lifetime ecstasy use on aggressive behaviour among users with low self-control
Romero-Martínez *et al*., 2019	Cross-sectional	102:63 IPVAW perpetrators and 39 controls (with no criminal history of IPVAW)	MDMA	Intimate partner violence, Interview questions about the IPVAW perpetration (i.e. type of aggression and severity of injuries)) and risk of reoffending assessed by the Spousal Assault Risk Assessment (SARA). Official records from the Spanish Government.	No differences between groups in MDMA and anabolic steroid consumption. Previous criminal record (delinquency without violence) related to cannabis, heroin and MDMA use.
Scott *et al*., 2013	Mixed and Correlational	56: 35 ecstasy users were compared with 21 who abstained	Ecstasy	Aggressive symptoms, The hostility scale from the Brief Psychiatric Rating Scale	In the days following use, ecstasy consumption was not associated with mood symptoms or clinician-rated subacute depressive, anxiety, or aggressive symptoms. Hours and sleep quality were associated with lowered mood and increased psychological distress.
Vaughn *et al*., 2015	Cross-sectional	19 067 non-institutionalised U.S. residents	MDMA	Criminal and Violent Behaviour, The antisocial personality disorder module of the Alcohol Use Disorder and Associated Disabilities Interview Schedule––DSM-IV version (AUDADIS-IV). Two additional dichotomous measures of involvement in any of the criminal and violent behaviours included.	MDMA users were significantly more likely to be involved in all criminal and violent behaviours (even when controlling for sociodemographic factors, parental antisocial and substance use characteristics, lifetime substance use and psychiatric morbidity) Gender differences: Male MDMA users were significantly more likely to enact violence. Female MDMA users were significantly less likely to commit acts of violence and had a higher prevalence of engaging in criminal and violent acts (except injuring someone in a fight) compared to male non-users, even after controlling for confounding variables
Verheyden *et al*., 2002	Parallel group	80: 40 participants who reported taking MDMA; 40 participants who reported using illicit substances excluding MDMA (polydrug controls).	MDMA	Trait Aggression and acute mood, Aggression Questionnaire and Aggression rating scale	From day 0 to day 4, an increase in self-rated aggression scores was found among MDMA users; the reverse was seen in the control group. In males: change in aggression correlated with the amount of MDMA taken. No significant differences between groups for trait aggression.
Verheyden *et al*., 2003	Descriptive cross-sectional	110 ex-users who used to take MDMA regularly who had not taken MDMA for at least 1 year (average 3 years)	MDMA, LSD	Anger and aggression, Multidimensional Anger Inventory and the Aggression Questionnaire	Years of MDMA use correlated significantly with BDI score, total aggression score, A.Q anger scale score, A.Q physical aggression scale score, and MAI frequency of anger scale score. Positive correlation between A.Q physical aggression and frequency and amount of MDMA used. Higher total aggression, physical aggression, anger and frequency of anger scores were seen in participants with a longer period of MDMA use LSD frequency of use and duration of use were higher in the mental health group.

The sample sizes of included studies ranged from 30 to 19,067 participants. A majority of the studies (*N* = 10) were conducted in Europe. Several studies included only males (*N* = 9) and did not report on ethnicity (*N* = 10). Sixteen studies focused on ages 16–50 (Gerra *et al*., 2000; Gerra *et al*., 2001; Verheyden *et al*., 2002; Verheyden *et al*., 2003; Bond *et al*., 2004; Curran *et al*., 2004; Hoshi *et al*., 2004; Hendrickson and Gerstein, 2005; Hoshi *et al*., 2006; Hoshi *et al*., 2007; Reid *et al*., 2007; Feingold *et al*., 2008; Scott *et al*., 2013; Vaughn *et al*., 2015; Walsh *et al*., 2016; Romero-Martínez *et al*., 2019). However, one study included participants over the age of 50 (Thiessen *et al*., 2018). Of the 17 studies, 14 focused on MDMA (Gerra *et al*., 2000; Gerra *et al*., 2001; Verheyden *et al*., 2002; Verheyden *et al*., 2003; Bond *et al*., 2004; Curran *et al*., 2004; Hoshi *et al*., 2004; Hendrickson and Gerstein, 2005; Hoshi *et al*., 2006; Hoshi *et al*., 2007; Reid *et al*., 2007; Scott *et al*., 2013; Vaughn *et al*., 2015; Romero-Martínez *et al*., 2019). Out of the three non-MDMA studies, only one study (Thiessen *et al*., 2018) assessed classic psychedelics as the main substance of research focus whereas the other two grouped hallucinogens into a broad category encompassing both classic and non-classic psychedelics (Feingold *et al*., 2008; Walsh *et al*., 2016). Most studies ascertained aggression or intimate partner violence (IPV) as outcomes, followed by criminal behaviour and aggressive cognitive bias. There is substantial heterogeneity concerning the instruments employed to measure these outcomes, as outlined below.

Included studies were grouped into three categories based on their outcome measures and results: the relationship between classic psychedelics and aggression, MDMA and criminality, and lastly, MDMA and aggression. A summary of the results can be found in Table 2.

**Table 2.** Summary of results

Relationship between:	Studies **supporting** an increase in outcome measures following use	Studies **not supporting** an increase in outcome measures following use
MDMA use **and** Anger and/or Aggression	7/14	3/14
MDMA use **and** Aggressive cognitive bias	2/14	2/14
MDMA use **and** *IPV	1/14	No Research
MDMA use **and** Criminal/violent behaviour	2/14	1/14
Classic Psychedelics/Hallucinogens use **and** Anger and/or Aggression	No Research	No Research
Classic Psychedelics/Hallucinogens use **and** Aggressive cognitive bias	No Research	No Research
Classic Psychedelics/Hallucinogens use **and** *IPV	1/3	2/3

* IPV, Intimate Partner Violence.

Psychedelic/MDMA use (Independent Variable).

Anger and/or Aggression, Aggressive cognitive bias, IPV (Dependent Variable).

### Classic psychedelics

One of the important predictors of IPV is reported to be substance use (Foran and O’Leary 2008), but the association between substance use and violence varies across each class of drugs. In a previous study, inmates who met criteria for lifetime hallucinogen use disorder (HUD) (*n* = 22) were less than half as likely to be arrested for perpetrating IPV compared to those without lifetime hallucinogen use disorder (*n* = 280) (Walsh *et al*., 2016). These findings suggest that hallucinogenic drugs may be protective against IPV. The use of hallucinogens also predicted reduced arrest for IPV independently (*β* = −0.54, SE = 0.20, χ2 = 7.19, exp(B) = 0.58, *p* < 0.01) as well as when controlling for covariates (*β* = −0.48, SE = 0.23, χ2 = 4.44, exp(B) = 0.62, *p* < 0.05) (Walsh *et al*., 2016). Contrary to these findings, a study of an at-risk community sample of men (*n* = 150) found that hallucinogen use had the largest effect on IPV (*η* ^2^ = .09) compared to other hard drugs, even after controlling for antisocial personality (Feingold *et al*., 2008).

Thus, published studies suggest that hallucinogens potentially have some protective effects against aggressive behaviours. However, two of the three studies combined classic psychedelics with other hallucinogens making it challenging to attribute this potential protective effect to one drug class. The only study (Thiessen *et al*., 2018) that reported specifically on classic psychedelics found that a lifetime history of classic psychedelic use (psilocybin and LSD) was inversely related to IPV perpetration in males (odds ratio = 0.42, *p* < 0.05). Interestingly, male psychedelic users reported better emotion regulation, which mediated the relationship between psychedelic use and IPV.

### Gender differences

No concrete conclusions can be drawn regarding gender differences in relation to psychedelic use and aggression. Two of the three hallucinogen studies included only males in their sample (Feingold *et al*., 2008; Walsh *et al*., 2016). Nevertheless, one study found that lifetime history of classic psychedelic use was inversely related to IPV perpetration in males (odds ratio = 0.42, *p* < 0.05) but not in females (odds ratio = 1.11; *p* > 0.05) (Thiessen *et al*., 2018).

### Instruments of measurement

Variability in IPV data collection methods may influence the definition of IPV and its related components. One study used various methods (e.g. self-reports, partner reports, interviewer ratings, behavioural assessment) to gather information on injuries and physical and psychological aspects of IPV (Feingold *et al*., 2008). In contrast, another used the revised short-form Conflicts Tactics Scale to measure couples psychological and physical aggression and pro-social negotiation tactics (Thiessen *et al*., 2018).

### MDMA

#### MDMA and criminality

We identified four studies (Hendrickson and Gerstein, 2005; Reid *et al*., 2007; Vaughn *et al*., 2015; Romero-Martínez *et al*., 2019) investigating potential associations between MDMA use and IPV, criminality and violent behaviour. The first study had a sample of 17,558 subjects consisting of arrestees and a matched sample of men who had not been arrested. This team found that among arrestees, ecstasy users compared to non-ecstasy users, were less likely to be charged with assault (OR = 0.682, *p* < 0.10) or burglary (OR = 0.498, *p* < 0.05) but more likely to be charged with drug-related crimes (OR = 1.29, *p* < 0.05) (Hendrickson and Gerstein, 2005). The second study (*n* = 102) reported that among male perpetrators of IPV against women, MDMA use was related to the existence of a previous criminal record without violence (Romero-Martínez *et al*., 2019). The third study (*N* = 19,067) found that, compared to non-MDMA users, MDMA users participated significantly more in criminal and violent behaviour, even after controlling for covariates such as lifetime substance use, sociodemographic factors and parental antisocial behaviours and substance use characteristics (Vaughn *et al*., 2015).Lastly, data collected from 260 ecstasy users showed higher levels of violent behaviour among users with high levels of lifetime ecstasy use (Reid *et al*., 2007).

#### MDMA and aggression: Longevity of effects

Acute MDMA use is commonly associated with elevated mood, pro-social behaviour and enhanced empathy (Vollenweider *et al*., 1998). However, the rapid release of serotonin combined with the inhibition of tryptophan hydroxylase (TPH) can result in serotonin depletion in the long term (Schmidt *et al*., 1986; McKenna and Peroutka, 1990). Low serotonin levels have been associated with depression and can modulate human aggression (da Cunha-Bang and Knudsen, 2021). Several studies have investigated the impact MDMA has on aggression, both immediately and in the days following consumption. Three of the reviewed studies found no significant difference between MDMA users and control groups on trait aggression as measured by the Aggression Questionnaire (A.Q) (Verheyden *et al*., 2002; Curran *et al*., 2004; Hoshi *et al*., 2004). However, four studies (including the three previously discussed), did find that aggression, as measured by self-report, increased on day four post-use (Verheyden *et al*., 2002; Curran *et al*., 2004; Hoshi *et al*., 2004; Hoshi *et al*., 2006). This is further supported by objective measures of aggression where MDMA users were faster at responding (Curran *et al*., 2004) and recognising (Hoshi *et al*., 2006) aggressive sentences compared to controls on day four post-use.

There is contradictory evidence regarding whether increased aggression following MDMA use is transient or persists. For example, Curran and colleagues (Curran *et al*., 2004) reported that self-rated aggression in ecstasy users had reverted to the same level as controls by day seven post-use. Similarly, Hoshi and colleagues (Hoshi *et al*., 2007), found that habitual ecstasy users abstinent for an average of two weeks did not report increased aggression compared to current users, polydrug users and drug-naïve controls. Furthermore, Gerra *et al*. (2000) showed that ‘direct aggressiveness’ scores on the Buss-Durkee Hostility Inventory (BDHI) decreased significantly in ecstasy users abstinent for 12 months, compared to their three weeks of abstinence scores (*df* = 1;28; *F* = 9.74; *p* < .05). However, in another study, scores from the Direct Aggression and Irritability scales of the BDHI (*t* = 3.87, df = 38, *P* < .001; *t* = 3.51, df = 38, *P* < .001) and aggressive responses to a laboratory task (*F* = 20.74, df = 76, *P* < .001) were significantly higher in ecstasy users who had been abstinent for three weeks compared to controls (Gerra *et al*., 2001). One caveat to consider regarding this study is that ecstasy users were recruited from a drug addiction service and some reported personality disorders and dysphoria. Thus, aggressive responding may be linked to other psychological problems that may have predated ecstasy use, and given that the control group was not matched on these factors, it is difficult to draw concrete conclusions.

Further investigations into the longevity of effects of MDMA were explored in data from 110 ex-MDMA users which were divided into two groups based on their reason for quitting: 1.mental health or 2. circumstantial reasons (Verheyden *et al*., 2003). There was a relationship between years of MDMA use and anger and aggression. In the mental health group, years of use correlated with the following scale scores: the Multidimensional Anger Inventory’s (MAI) frequency of anger (*r* = 0.55, *p* < 0.005) and aggression (total score) (*r* = 0.68, *p* < 0.001), and the Aggression Questionnaire’s (A.Q) anger (*r* = 0.51, *p* < 0.01) and physical aggression (*r* = 0.53, *p* < 0.005). In the circumstantial group, physical aggression scores on the A.Q had a positive correlation with frequency (*r* = 0.60, *p* < 0.01) and amount of MDMA use (*r* = 0.64, *p* < 0.01) (Verheyden *et al*., 2003). It is important to note that the frequency (*U* = 124.00, *p* < 0.005) and duration (*U* = 89.00, *p* < 0.01) of LSD use were higher among participants in the mental health group compared to the circumstantial group. Furthermore, data collected from 260 ecstasy users in the United States, found individuals with high levels of lifetime ecstasy use had higher levels of violent and aggressive behaviour (Reid *et al*., 2007). Based on this finding, the authors concluded that there is a relationship between high levels of aggressive and violent behaviour and high prevalence of lifetime ecstasy use. Additionally, high levels of lifetime ecstasy use was found to impact individuals with high self-control more than those with low self-control (i.e. more aggression was observed in individuals with high self-control) (Reid *et al*., 2007).

In a correlational study (Scott *et al*., 2013) with a sample of 56 participants (35 ecstasy users and 21 abstained ecstasy users), quality and hours of sleep were associated with lower mood and increased psychological distress. Ecstasy use was not found to be related to self-reported mood symptoms or clinician-rated subacute symptoms of depression, anxiety, or aggression in the days following use.

Finally, Bond *et al*. (2004) employed a tryptophan depletion/enhancement strategy to investigate the effects of 5-HT on ‘angry responding’ which was based on the participants’ reaction towards a story-based task. Trait anger and aggression were measured by administering the A.Q (Buss and Perry, 1992) and the MAI (Siegel, 1986) in current MDMA users who abstained for three weeks, ex-users abstinent for longer than one year and non-MDMA substance users. All participants processed angry sentences faster than non-angry ones, and this relationship was strongest among current MDMA users (*r* = 0.40, *P* = 0.028). Furthermore, ex-MDMA users had higher scores than controls on aggression, as seen from their total scores on the A.Q (F2,91 = 3.72, *P* < 0.03) (Bond *et al*., 2004).

#### Gender differences

Half of the MDMA studies included only men (Gerra *et al*., 2000; Gerra *et al*., 2001; Verheyden *et al*., 2003; Bond *et al*., 2004; Hendrickson and Gerstein, 2005; Hoshi *et al*., 2007; Romero-Martínez *et al*., 2019) and the few studies that included both genders were underpowered to complete gender-based analyses (Curran *et al*., 2004; Hoshi *et al*., 2004).

Four studies analysed the data between male and female MDMA users (Verheyden *et al*., 2002; Hoshi *et al*., 2006; Reid *et al*., 2007; Vaughn *et al*., 2015). In one study, self-rated trait aggression scores on day 0 and day 4 post-MDMA use did not differ between males and females (Verheyden *et al*., 2002). Additionally, there were some indications of gender differences in residual responses to MDMA, where the amount of MDMA consumed correlated positively with increased aggression scores in males at mid-week (*r* = 0.66, *P* < 0.005), but not in females. Based on the analysis of MDMA use patterns (i.e. dose, frequency of use and years of use), men had taken more MDMA tablets over their lifetime than women (*t*_22_= –2.76, *P* < 0.05)(Verheyden *et al*., 2002). These results are similar to those reported by Vaughn *et al*. (2015) who found that male, but not female, MDMA users were significantly more prone to commit acts of violence (AOR = 1.73, 95% CI = 1.51–2.00; AOR = 0.77, 95% CI = 0.63–0.94). Additionally, compared to female non-users, female MDMA users were significantly less likely to commit acts of violence and compared to male non-users, had a higher prevalence of engaging in criminal and violent acts (except injuring someone in a fight), even after controlling for confounding variables (Vaughn *et al*., 2015). Additionally, Reid *et al*. (2007) found that males exhibited less aggressive behaviour compared to females when looking at the impact of lifetime ecstasy use on aggressive behaviour among users with low self-control. In contrast, Hoshi *et al*. (2006) found no differences in aggressive interpretative bias and self-rated aggression in male and female MDMA users.

#### Instruments of measurement

Among MDMA studies, half of the studies (*N* = 7/14) administered the Aggression Rating Scale (ARS) (Bond and Lader, 1986) and/or the A.Q (Buss and Perry, 1992) to investigate participant aggression/anger (Verheyden *et al*., 2002; Verheyden *et al*., 2003; Bond *et al*., 2004; Curran *et al*., 2004; Hoshi *et al*., 2004; Hoshi *et al*., 2006; Hoshi *et al*., 2007). Two studies (Gerra *et al*., 2000; Gerra *et al*., 2001) utilised the BDHI (Buss and Durkee, 1957) to quantify and characterise aggressiveness (direct, indirect, verbal; irritability; negativism; resentment; suspiciousness; guilt). Vaughan and team (Vaughn *et al*., 2015) administered the antisocial personality disorder module from the Alcohol Use Disorder and Associated Disabilities Interview Schedule (Grant *et al*., 2003) to gather data on violent behaviour in addition to using dichotomous questions to investigate involvement in criminal behaviour. One study used the MAI (Verheyden *et al*., 2003), and Reid *et al*. (2007) created an additive scale that investigated aggressive behaviours. Lastly, Scott *et al*. (2013) used Brief Psychiatric Rating Scale (BPRS) (Ventura *et al*., 1993) items to measure aggression.

## Discussion

The current narrative review investigated the association between psychedelic use and violent/aggressive behaviour among lifetime psychedelic users. Overall, it is difficult to draw any firm conclusions from the published data because of several shortcomings including lack of standardised measures, non-specific drug categorisation and failure to control for potential confounders or to provide details on gender differences, among other issues. These are outlined below.

### Gender differences across studies

As highlighted in the results, it is difficult to draw any firm conclusions regarding gender differences in violent/aggressive behaviour amongst psychedelic users. Several reviewed studies included only males or were underpowered to detect gender differences. A small portion of the MDMA studies did investigate gender differences, but the findings were variable. This variation may be due to the diversity in measurements utilised to assess aggression across the various studies making comparisons difficult. For example, a meta-analysis found that in comparison to naturalistic studies, experimental studies were less likely to find increased aggression in men and gender differences were greater for certain forms of aggression (e.g. physical vs. verbal aggression) (Knight *et al*., 2002). Men tend to express physical, overt and direct aggression, while relational and indirect aggression is more often found in women (Im *et al*., 2018). The aggression scales used in seven of the fourteen MDMA studies investigated trait aggression which is generally more common in men. Administering aggression scales that assess various types of aggression would provide a more comprehensive understanding of gender differences and help to establish a greater degree of accuracy.

### Polysubstance use

An important confounding factor across many of the reviewed studies is the use of other substances. In 13 of the 14 MDMA studies (Gerra *et al*., 2000; Gerra *et al*., 2001; Verheyden *et al*., 2002; Verheyden *et al*., 2003; Bond *et al*., 2004; Curran *et al*., 2004; Hoshi *et al*., 2004; Hendrickson and Gerstein, 2005; Hoshi *et al*., 2006; Hoshi *et al*., 2007; Reid *et al*., 2007; Scott *et al*., 2013; Romero-Martínez *et al*., 2019) and two of the three hallucinogenic/classic psychedelic studies (Feingold *et al*., 2008; Walsh *et al*., 2016), most, if not all, participants were episodic or frequent users of alcohol and other substances of abuse. Some studies tried to mitigate this by asking participants to abstain. For example, Bond *et al*. (2004) requested abstaining from cannabis; however, abstinence from habitual cannabis can be associated with withdrawal symptoms, which might exacerbate aggressive tendencies. Similarly, participants in another study met the criteria for other substance use disorders, including alcohol use disorder, which is known to influence aggression (Walsh *et al*., 2016). In some of the MDMA studies, the researchers mitigated the effects of polysubstance use by excluding participants who had dependencies on other substances (Gerra *et al*., 2000; Gerra *et al*., 2001), or by controlling for confounding variables in their analyses as seen in two MDMA studies (Hoshi *et al*., 2007; Vaughn *et al*., 2015) and the classic psychedelic study (Thiessen *et al*., 2018). However, these strategies are not adequate to fully delineate the effects of psychedelics on aggression/violence and criminality from the impact of other drugs. Polysubstance users vary in the number and category of substances they use and the frequency, dose and duration of use. The level of impact of each substance on violent and/or aggressive behaviour varies across categories of substances (Boles and Miotto, 2003). For example, more studies suggest that cocaine use increases risk of aggression compared to the mixed findings associated with cannabis use (Boles and Miotto, 2003; Zhong *et al*., 2020). Variability in usage behaviour, in addition to the variability in the magnitude of impact from each substance on aggressive/violent behaviour, makes it challenging to draw any conclusions from a polysubstance sample. Moreover, some polysubstance users may have started to use various substances to intentionally control the cognitive, emotional or behavioural side effects experienced from a single substance (Kataja *et al*., 2018; Foundation, 2021). For instance, an individual may be experiencing aggressive behaviour due to their MDMA use and may start to use cannabis in an attempt to ameliorate this effect. It is also important to note that a study of a university student sample found that polysubstance users reported higher physical and verbal aggression than mono-substance users (Steele and Peralta, 2020). The presence of multiple substances and the interaction between these substances may be the reason for this finding. Generally, interaction effects and developmental, psychosocial and personality characteristics associated with substance use and aggression make such disaggregation challenging. Furthermore, some psychedelics may directly affect the use and dependence of other drugs. For example, ayahuasca has been found to be effective in treating substance dependence (Frecska *et al*., 2016).

### Mental health

Further complexities in interpreting the reviewed literature lie in associations between previous aggressive behaviour and emotion regulation (Gerra *et al*., 2001; Thiessen *et al*., 2018). For example, the decreased odds of reporting IPV among male psychedelic users compared to males with no psychedelic history may be attributed –in part - to emotion regulation (Thiessen *et al*., 2018). When controlling for emotion regulation, the negative association between psychedelic use and IPV was reduced (*b* = −0.09, SE = 0.04, *p* > 0.05). These results suggest that emotion regulation influences the relationship between lower perpetration of IPV and psychedelic use. In contrast, another study reported that hallucinogen use (both classical and non-classical psychedelics) had the largest effect on IPV compared to other hard drugs, even after controlling for antisocial personality (Feingold *et al*., 2008).

Looking at MDMA, pre-existing personality traits that influence an individual’s aggressive behaviour may be a factor in mediating aggression in the context of MDMA use (Gerra *et al*., 2001). Research also suggests that the likelihood of substance use may be related to pre-existing anger levels: those with high anger levels are more likely to use drugs (Serafini *et al*., 2016). This theory is supported by Bond *et al*. (2004) who found no difference in processing time of angry versus non-angry sentences between current MDMA users abstinent for three weeks, ex-users abstinent for longer than one year and non-MDMA substance users. All participants processed angry sentences faster than non-angry ones. These results suggest that individuals with high pre-existing anger levels may be more prone to use MDMA thus, reports of increased anger following MDMA use may not directly be related to the use of MDMA (Bond *et al*., 2004). Furthermore, Reid *et al*. (2007) found that self-control may also mitigate the influence that ecstasy use has on aggression. Those with high self-control were more aggressive at high levels of lifetime ecstasy use. In contrast, those with low self-control were found to be less affected by high levels of lifetime ecstasy use, with little change in aggressive behaviour (Reid *et al*., 2007).

### Challenges with assessment measures

As outlined in the results, there is heterogeneity among studies regarding the assessment measures used to evaluate aggression. This makes it challenging to compare observations across studies. In addition, a majority (*N* = 14/17) of the reviewed studies relied on self-report when collecting substance use history and did not employ confirmatory, objective lab tests such as urine or hair analysis (Verheyden *et al*., 2002; Verheyden *et al*., 2003; Bond *et al*., 2004; Curran *et al*., 2004; Hoshi *et al*., 2004; Hendrickson and Gerstein, 2005; Hoshi *et al*., 2006; Reid *et al*., 2007; Feingold *et al*., 2008; Scott *et al*., 2013; Vaughn *et al*., 2015; Walsh *et al*., 2016; Thiessen *et al*., 2018; Romero-Martínez *et al*., 2019). Data collected through self-report may be biased due to social desirability or recall bias which can jeopardise data accuracy and may explain the mixed findings observed (Althubaiti, 2016).

Cohesion across studies on which instruments are used would enhance comparability between results. Moreover, each instrument has certain limitations and strengths which need to be considered before being employed in the study. For instance, the BDHI can measure the level of aggression influenced by the environment, life events and pharmacological use (Buss and Durkee, 1957), but misses out on assessing areas of aggression such as physical, verbal, anger and hostility, which are directly measured by the Aggression Questionnaire (Buss and Perry, 1992).

### Variability in duration, frequency and dose

There was substantial variability between studies regarding the duration and frequency of use for the drugs that were assessed. For example, in one study MDMA users were defined as having taken MDMA on at least 20 occasions during the previous year (Verheyden *et al*., 2002) while in another study, MDMA users had to have taken MDMA at least once a month with a minimum of 25 uses (Hoshi *et al*., 2007).

Additionally, a prevalent issue in clinical practice regarding substance use treatment and clinical effects following drug use is drug impurity (Shesser *et al*., 1991). It is difficult to determine the actual dosage, level of purity and chemical make up of street drugs which is only further complicated by the fact that most participant samples consist of individuals who used multiple substances. There is heterogeneity in the make up of drugs and the actual dosage of pure substance used by each individual. In one study, participants reported using 1–2 pills, only on weekend nights (Gerra *et al*., 2000). In contrast, the mean number of tablets per session of use was found to be 2.71 among MDMA participants of another study (Curran *et al*., 2004). This phenomenon may explain the mixed findings across MDMA studies given that other amphetamine analogues and other compounds, such as caffeine, have been found in ecstasy tablets (Wolff *et al*., 1995).

Lastly, further research should explore the extent to which the duration and frequency of psychedelic use impacts aggressive and violent behaviour in MDMA users. The ability to recognise fearful faces on the fourth day following use has been demonstrated to have a negative relationship between duration (*r* = −0.54, *P* = 0.048) and amount (*r* = −0.58, *P* = 0.031) of ecstasy use (Hoshi *et al*., 2004). This means that more ecstasy use at baseline and an extended history of use was associated with worse fear recognition accuracy score on day four. Reid and colleagues (Reid *et al*., 2007) also found a linear relationship between the prevalence of committing violent acts and the number of ecstasy pills ever used.

## Limitations and future directions

There is abundant room for further progress in exploring associations between psychedelic use and aggressive/violent behaviour. This research is especially important given the growing interest from the research community and the general public regarding psychedelics as potential treatments for mental health and addictions. There are several limitations regarding the reviewed literature. First, in several of the reviewed studies, ‘hallucinogen’ was used as a broad category to include several serotonergic psychedelics. Early research investigating the use of classic psychedelics for therapeutic benefits among incarcerated individuals demonstrated positive personality changes (Arendsen-Hein, 1963; Leary, 1969) and enhanced empathy, communication and insight (Tenenbaum, 1961). However, rigorous scientific methodology was not employed in these studies. Of the three non-MDMA studies reviewed (Feingold *et al*., 2008; Walsh *et al*., 2016; Thiessen *et al*., 2018), only one distinguished classic serotonergic psychedelics (i.e. LSD, psilocybin) from non-classic psychedelics (i.e. PCP, MDMA) (Thiessen *et al*., 2018). As discussed in the introduction, the mechanism of action of classic psychedelics and empathogens differ (Nichols, 1986; Garcia-Romeu *et al*., 2016). Grouping these drugs into one category limits our understanding of the impact that each one may have on aggressive/violent behaviour and precludes specific conclusions from being drawn. Studies that compare the effects of using classic versus non-classic psychedelics are needed to understand the extent to which agents from each class impacts aggressive behaviours. Future studies should differentiate individual substances and their relation to aggression/violence.

Second, several studies included participants who were polysubstance users (Gerra *et al*., 2000; Gerra *et al*., 2001; Verheyden *et al*., 2002; Verheyden *et al*., 2003; Bond *et al*., 2004; Curran *et al*., 2004; Hoshi *et al*., 2004; Hendrickson and Gerstein, 2005; Hoshi *et al*., 2006; Hoshi *et al*., 2007; Reid *et al*., 2007; Feingold *et al*., 2008; Scott *et al*., 2013; Walsh *et al*., 2016; Romero-Martínez *et al*., 2019). Future research projects should focus on controlling for confounding variables such as psychiatric disorders, polysubstance use, personality characteristics and pre-existing behaviours to explore the association between psychedelic use and aggressive/violent behaviour. Third, most of the studies relied on self-report regarding the use of recreational drugs. Without proper laboratory analysis, we cannot be certain about the purity or dose of the substances being consumed. Fourth, the type and extent of aggression/IPV varied substantially across studies, with some research focusing on violent criminal activity and others focusing on objective measures (e.g. interpretative bias tasks). While it could be argued that aggressive interpretative bias may underlie violence, it is difficult to conclude the role psychedelics play in this relationship and it has yet to be rigorously investigated. Fifth, there was variation in measurements of aggression, with few studies employing objective and subjective measures (Bond *et al*., 2004; Curran *et al*., 2004; Hoshi *et al*., 2004; Hoshi *et al*., 2006; Hoshi *et al*., 2007). To develop a complete picture on the extent of influence psychedelic use has on aggression, additional studies that include multidimensional and validated measures of aggression are needed. Lastly, more longitudinal research is needed to understand the relationship between trait aggression and prolonged cessation of use and whether increases in aggression following use is transient or long-lasting.

A number of studies from our initial search were excluded as they had not employed standardised questionnaires or because the outcome variables did not directly relate to violence or aggression (Wu *et al*., 2006; Hughes *et al*., 2008; Håkansson and Berglund, 2012; Raznahan *et al*., 2013; Carbonaro *et al*., 2016; Hendricks *et al*., 2018; Jones and Nock, 2022). One of these studies found that psychedelic use (Ayahuasca, DMT, LSD, mescaline, peyote, psilocybin) was associated with lower odds of property crime, theft, assault and violent crime compared to other illicit substance use (Hendricks *et al*., 2018). Building on this, another study reported significantly lowered odds of seven crime arrest outcomes (larceny, burglary, robbery, simple assault/battery, serious violence, driving under the influence and miscellaneous crimes) among participants with a lifetime history of psilocybin use (Jones and Nock, 2022). Although these studies are informative, they were excluded because the data was collected from The National Survey on Drug Use and Health and standardised anger/aggression measures were not utilised. Most psychedelic drugs are presumed to demonstrate relatively good physical and psychological safety profiles, in addition to therapeutic potential for mental health and substance use disorders, leading to a renewed interest in their potential therapeutic use (Perkins *et al*., 2021).

It is important to consider the difference between ‘hostility’ and ‘aggression’. Violence is often seen as an extreme form of aggression (Allen and Anderson, 2017). In reviewing the literature, there is strong evidence to suggest an increased risk of aggressive behaviour and violent crimes is associated with the use of many other drugs of abuse (Pihl and Peterson, 1995; Boles and Miotto, 2003). We were interested in the overt and observable anger and not hostility which can be defined as an attitude (Eckhardt *et al*., 2004). Finally, our study was limited to research articles published between 2000 and 2022. There are numerous studies conducted before the early 2000s that investigated relationships between psychedelics and aggression. However, we were interested in more recent trials and thus excluded studies done before the year 2000.

## Conclusions

Findings from studies on associations between MDMA use and aggressive/violent behaviour are conflicting. The data on classic psychedelic use and aggression is limited, and no definitive conclusions can be drawn. Thus far, classic psychedelic use has not been shown to be associated with increased violent offences such as IPV. Variability in sample characteristics, instruments of measurement and confounding factors may explain some of the inconsistencies in the current literature. Researchers should ensure they have robust and diverse sample sizes and consider implementing more rigorous methods of analysis into future studies.
